# Do Orthopedic Surgeons or Neurosurgeons Detect More Hip Disorders in Patients with Hip-Spine Syndrome? A Nationwide Database Study

**DOI:** 10.3390/brainsci11040485

**Published:** 2021-04-11

**Authors:** Tsung-Cheng Yin, Adam M. Wegner, Meng-Ling Lu, Yao-Hsu Yang, Yao-Chin Wang, Woon-Man Kung, Wei-Cheng Lo

**Affiliations:** 1 Department of Orthopedic Surgery, Kaohsiung Chang Gung Memorial Hospital, Chang Gung University College of Medicine, Kaohsiung 83301, Taiwan; tsongchenyin@gmail.com (T.-C.Y.); lumengling@gmail.com (M.-L.L.); 2 OrthoCarolina, Winston-Salem, NC 27106, USA; adamwegner@gmail.com; 3 Department of Traditional Chinese Medicine, Chang Gung Memorial Hospital, Chiayi 61363, Taiwan; r95841012@ntu.edu.tw; 4 Health Information and Epidemiology Laboratory of Chang Gung Memorial Hospital, Chiayi 61363, Taiwan; 5 School of Traditional Chinese Medicine, College of Medicine, Chang Gung University, Taoyuan 33302, Taiwan; 6 Department of Emergency, Min-Sheng General Hospital, Taoyuan 33044, Taiwan; vkwang8888@yahoo.com.tw; 7 Graduate Institute of Injury Prevention and Control, College of Public Health, Taipei Medical University, Taipei 11031, Taiwan; 8 Department of Exercise and Health Promotion, College of Kinesiology and Health, Chinese Culture University, Taipei 11114, Taiwan; nskungwm@yahoo.com.tw; 9 Master Program in Applied Epidemiology, College of Public Health, Taipei Medical University, Taipei 11031, Taiwan

**Keywords:** hip-spine syndrome, total hip arthroplasty, orthopedic surgeon, neurosurgeon, Taiwan National Health Insurance Research Database

## Abstract

*Background*: Disorders of the hip and lumbar spine can create similar patterns of pain and dysfunction. It is unknown whether all surgeons, regardless of orthopedic or neurosurgery training, investigate and diagnose concurrent hip and spine pathology at the same rate. *Methods*: Data were retrieved from Taiwan’s National Health Insurance Research Database (NHIRD). Enrolled patients were stratified into hip and spine surgery at the same admission (Both), hip surgery before spine surgery (HS), or spine surgery before hip surgery (SH). The SH group was further subdivided based on whether spine surgery was performed by an orthopedic surgeon (OS) or neurosurgeon (NS), and differences in preoperative radiographic examinations and diagnoses were collected and analyzed. *Results*: In total, 1824 patients received lumbar spine surgery within 1 year before or after hip replacement surgery. Of these, 103 patients had spine and hip surgery in the same admission (Both), 1290 patients had spine surgery before hip surgery (SH), and 431 patients had hip surgery before spine surgery (HS). In the SH group, patients were categorized into spine surgery by orthopedic surgeons (OS) (*n* = 679) or neurosurgeons (NS) (*n* = 522). In the SH group, orthopedic surgeons investigated hip pathology with X-rays more often (52.6% vs. 38.1%, *p* < 0.001) and diagnosed more cases of hip disease (43.6% vs. 28.9%, *p* < 0.001) than neurosurgeons. *Conclusions*: Of patients in Taiwan’s NHIRD who had concurrent surgical degenerative hip and lumbar spine disorders who had spine surgery before hip surgery, orthopedic surgeons obtained hip images and made hip-related diagnoses more frequently than did neurosurgeons.

## 1. Introduction

Osteoarthritis (OA) is the most common musculoskeletal disease of the elderly and the most common cause of musculoskeletal-related disabilities worldwide. Approximately 1.2 million physician office visits per year in the United States are attributed to symptoms of degenerative lumbar spinal stenosis, and it is the most frequent indication for spinal surgery in patients >65 years of age [1]. Symptomatic hip OA is also very common, as it has been diagnosed in 9.2% of adults ≥45 years old in the United States [2].

Not surprisingly, degenerative hip and lumbar spine pathologies may mimic each other. Buttock and back pain are traditionally associated with lumbar spine pathology, whereas groin pain is more often associated with hip pathology. However, pain from hip OA can localize to the groin (84%), buttocks (76%), anterior thigh (59%), posterior thigh (43%), anterior knee (69%), shin (47%), or calf (29%) [3,4]. Several studies also reported that compression of lumbar nerve roots may cause referred pain to the hip [5,6]. The likely explanation for the commonality in pain diagrams between the hip and lumbopelvic region is an overlap in innervation. The anterior joint capsule of the hip is innervated by the femoral and obturator nerves (L2~L4), while the posterior capsule is innervated by the sciatic and superior gluteal nerves (L4~S1) [7].

Since pathologies of the lumbar spine and hip are very common, they often coexist in the same patient. Hip-spine syndrome, the clinical scenario of concurrent hip OA and degenerative lumbar spinal stenosis, was first described by Offierski and MacNab in 1983 [8]. Lee at al. reported that 32.5% of 388 patients who underwent spinal surgery had significant hip pathology [9]. Because hip and spine pathologies can mimic each other, misdiagnoses of the etiology of pain in hip-spine syndrome do occur, which can result in inappropriate treatment [10]. Thus, treating physicians must order correct radiographic studies to assess both the lumbar spine and hip and correlate those findings with history taking and physical examination results to arrive at a correct diagnosis [3,8,11].

In general, neurological surgery residency provides more time and higher minimum requirements for spine surgery exposure across its minimum 6-year training length compared to orthopedic surgery residency and its minimum 5-year training period. However, training of orthopedic surgery residents focuses on the entire musculoskeletal system, including bone and joint pathophysiologies, musculoskeletal injuries, and rehabilitation, whereas there is no required training in the diagnosis or treatment of non-spine musculoskeletal conditions in neurosurgery training. As such, neurosurgery residents in North America perform almost four times as many spine procedures during residency as orthopedic residents [12]. The resident orthopedic and neurosurgery training systems in Taiwan are similar to those in Western countries.

Because of these differences in training and focus of these specialties, the purpose of this study was to evaluate whether orthopedic surgeons or neurosurgeons diagnose more hip degenerative disorders in patients with concurrent hip and spinal degenerative disorders undergoing surgery for both conditions within a one-year period.

## 2. Materials and Methods

### 2.1. Data Source

A nationwide cohort was collected using population-based data from the Taiwan National Health Insurance Research Database (NHIRD). Taiwan launched a compulsory National Health Insurance (NHI) program in 1995 that covers approximately 99% of Taiwan’s national population of 23 million. All inpatient and outpatient records, including patient characteristics such as sex, date of birth, date of admission, date of discharge, dates of visits, clinical diagnoses, procedures and medications, examinations, and expenditures, are collected with strict guidelines to provide a confidential dataset for research purposes.

This study was approved by the Research Ethics Committee of Chang-Gung Medical Foundation Institutional Review Board (no. 201801118B0C601). The Institutional Review Board waived the requirement for written informed consent from each of the patients involved since all data in the Taiwan NHIRD are deidentified.

### 2.2. Study Population

Patients undergoing specific hip arthroplasty and lumbar spine decompression or fusion procedures were selected using NHIRD procedural codes (see Table A1). Patients admitted for primary hip arthroplasty, including total hip arthroplasty (64162B) or partial hip arthroplasty (64170B), from January 1997 to December 2013, were identified from the Taiwan NHIRD. From that subset of patients, those who were 50~85 years old and who had received lumbar spinal surgery, including fusion surgery (83043B, 83044B, 83045B, or 83046B), laminectomy (83002C or 83003C), or lumbar discectomy (83024C) within 1 year before or after hip replacement surgery, were enrolled. All inclusion and exclusion procedure and diagnostic codes are listed in Table A1, Table A2 and Table A3. In order to select for patients undergoing surgery for lumbar or lumbosacral degenerative disease, patients receiving a cervical discectomy (83022c), procedures for spinal fracture (64160B), or with a diagnosis of a cervical spinal disorder (ICD-9 CM: 723*) or spinal trauma (ICD-9-CM: 805* or 806*) were excluded. Patients not seen in either an orthopedic or neurosurgery clinic within 6 months of their first surgery were also excluded.

Demographic data, hospital length of stay, surgeon specialty, and preoperative radiographic examination items between (1) patients who received hip and spine surgery at the same admission (Both), (2) hip surgery before spine surgery (HS), and (3) spine surgery before hip surgery (SH) were collected. Patients in the SH (spine surgery prior to hip surgery) group were further subdivided into (1) spine surgery performed by an orthopedic surgeon (OS) or (2) spine surgery performed by a neurosurgeon (NS).

### 2.3. Statistics

Descriptive statistics of the Both, HS, and SH groups were performed, including demographic data, hospital stay, Charlson Comorbidity Index (CCI), specialty of the surgeon, and preoperative X-ray examination items. Differences between the OS and NS groups were assessed using independent Student’s t-test for continuous variables and χ2 test for nominal variables. Significance was defined as *p* < 0.05 in two-tailed testing. All data statistical analyses were performed using SAS for Windows 9.3 (SAS Institute, Cary, NC, USA).

## 3. Results

**Figure 1:**
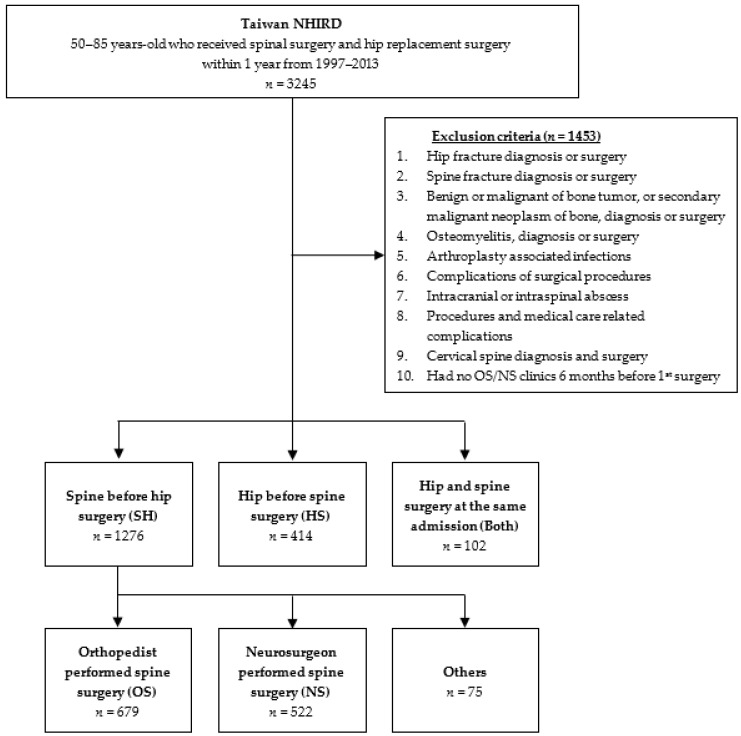
Flow diagram of inclusion and exclusion criteria for patient selection from the Taiwan National Health Insurance Research Database (NHIRD).

From the Taiwan NHIRD, 3245 patients aged 50~85 years old received spinal surgery within 1 year before or after hip replacement surgery in 1997 to 2013. Of these, 1453 patients were removed from the cohort based on the exclusion criteria. Among the remaining 1824 patients, 103 were categorized into the “Both” group, in which hip surgery and spine surgery were performed during the same admission; 1290 were categorized into the spine surgery before hip surgery (SH) group; and 431 had hip surgery performed before spine surgery, the (HS) group (Figure 1). The mean age, hospital stay, duration between admissions, CCI, and gender breakdown are shown in Table 1.

Numbers of patients who visited a clinic and had surgery by orthopedic or neurosurgeons are shown in Table 2. In the SH group, 53.2% (679) of first surgeries (spine surgery) were performed by an orthopedic surgeon (OS group) and 40.9% (522) were performed by a neurosurgeon (NS group).

Within 6 months of the first surgery, lumbar-spine radiographs were performed in 85.3% of the Both group, 60.1% in the HS group, and 82.1% in the SH group (Table 3). A kidney/ureter/bladder (KUB) X-ray was performed within 6 months of surgery for 7.8% in the Both group, 20.3% in the HS group, and 23.7% in the SH group. Pelvic and hip X-rays were performed in 71.6% of the Both group, 81.1% of the HS group, and 34.9% of the SH group.

In those who had hip surgery first (HS), 63.8% received a diagnosis of hip OA before surgery and 16.4% were diagnosed with hip osteonecrosis before surgery. Over half (51.9%) had a lumbar spinal disease-related diagnosis before their hip surgery. In those who had spinal surgery first (SH), 37.6% had a hip OA-related diagnosis, 3.5% had an osteonecrosis diagnosis, and 75.5% had lumbar spine-related diagnoses within 6 months before surgery (Table 3). In the HS group, 89.2% had a diagnosis of hip OA and 34.8% were diagnosed with hip osteonecrosis at first discharge. In the SH group, 98.8% had a diagnosis of a spine-related disease on discharge (Table 4).

In the OS group, 566 (83.4%) patients received an L-spine X-ray series and 357 (52.6%) received an L-spine X-ray combined with KUB or pelvic X-ray. This is in contrast to the NS group, in which 420 (80.5%) received an L-spine X-ray series, while significantly fewer NS patients (199; 38.1%) received an L-spine X-ray in addition to KUB or pelvic X-ray than OS patients (Table 5). The percentage of hip-related diagnoses was also higher in the OS group before the first surgery (43.6% vs. 28.9%, *p* < 0.001) (Table 6).

## 4. Discussion

Due to differences in training for specialty, one might hypothesize that orthopedic surgeons could detect more hip pathology than neurosurgeons for patients with concurrent hip and spine disorders. However, there was no previous scientific evidence to support this hypothesis. In this study of patients from Taiwan’s NHIRD with hip-spine syndrome who had hip arthroplasty within 1 year of spine surgery, significantly more patients were assessed using hip X-rays and were diagnosed with hip pathology before surgery if their spine surgery was performed by an orthopedic surgeon. This may have been due to differences in training and scope of practice of orthopedic surgeons versus neurosurgeons.

We believe that history taking, physical exams, and imaging to assess hip pathology should be considered in all lumbar spine evaluations. Clicking, snapping, pain, or loss of range of motion may indicate intra-articular hip pathology [4,13]. To rule out spine and hip conditions on initial screening radiographs, separate lumbar spine and pelvis plain radiographs are commonly conducted. Anteroposterior and lateral lumbar spine radiographs allow for identification of osteoarthritic changes in the apophyseal joints, disc height decrease, and neural foraminal narrowing. We included KUB radiographs in this study as the KUB radiograph provides visualization of the hip joint at a rate of 98.2% and is therefore often used to assess the hips [9]. One possible explanation for differences seen between specialties in evaluating and diagnosing hip pathology concomitant with a lumbar evaluation is that physical examinations and evaluations of hip pathology are included in orthopedic residency but are generally not part of neurosurgery residency.

There is controversy that should be addressed first in concurrent hip and spine disorders. In this study, significantly more patients first underwent spine surgery rather than hip surgery. We believe that diagnosing the primary pain generator should drive the decision on whether to address the lumber spine or hip first. It is essential to ensure that the patient understands that treatment of one condition can improve the activity level and possibly make the untreated condition more symptomatic. Patients should be informed prior to the first procedure that a second procedure may be necessary to alleviate their symptoms. McNamara et al. reported on 14 patients who underwent lower-extremity arthroplasty and were symptomatic with lumbar spinal stenosis after surgery [11]. Five initially presented with symptoms of both hip joint disease and spinal stenosis, whereas nine became symptomatic from spinal stenosis after their lower extremity reconstruction. Conversely, addressing one pathology may improve pain from the other. Of 170 patients who underwent total hip arthroplasty who also had concurrent low-back pain, 66.4% noted resolution of the low-back pain after hip arthroplasty, suggesting that management of the hip pathology may sometimes improve pain from lumbar spine pathology [14]. As in the first study however, 20% of those in whom low-back pain was not noted preoperatively developed pain within 1 year postoperatively, again suggesting that total hip arthroplasty can also exacerbate lumbar spine pathology [14].

In the present study, the number of patients in the SH group was three times higher than that in the HS group (1276 vs. 414). There are no large-scale studies reporting the percentages of hip or spine surgeries performed first for patients with concurrent degenerative hip and spine disorders. In the entire study cohort, there were fewer patients with a hip-related diagnosis than a spine-related diagnosis (1245 vs. 915). In the SH group, there were fewer preoperative hip-related diagnoses in both the OS and NS subgroups than lumbar spine-related diagnoses (OS: 43.6% vs. 72.3%; NS: 28.93% vs. 79.5%). This suggests that before spine surgery, hip pathology is possibly more easily missed than vice versa. This could also explain why there were more patients in the SH than the HS group. In Taiwan, nearly all hip arthroplasty surgeries and implants used for arthroplasty are covered by the NHI program; so hip arthroplasty surgeries need to be approved before surgery. But for spine surgeries, if a spinal implant is not required (e.g., for a laminectomy or discectomy) or spinal implants are not covered by the NHI, surgeries could be performed without the need for approval. This could be another reason there are more patients in the SH group than the HS group. The CCI was also higher at the first and second admissions in the HS group than in the SH group. Hip arthroplasty may be performed first in patients with hip-spine syndrome if the patient has more comorbidities.

There are several limitations of this study. Due to the structure of the database, details from medical charts, radiographic data, and operative reports could not be queried. Exact symptoms and results of the physical examination were also not captured. The severity of hip and lumbar spine disorders, possibly a key influence on the order that surgery occurred, was likewise unavailable. Other interventions for pain management or diagnosis, like hip injections or spinal epidural steroid injections before the surgical intervention, were not assessed. Exclusion criteria did not include peripheral vascular disease or diabetic peripheral neuropathy, which could exist in patients with leg pain. We tried to gather the most common spine and hip disease codes associated with surgery. The rate of spine-related diagnoses at discharge in patients who received spine surgery first was 98.8%, and that of hip-related diagnoses at discharge in patients who received hip arthroplasty first was 90.1% for hip OA and 34.8% for hip osteonecrosis, which were higher than the preoperative diagnosis rate. It is possible that physicians may choose non-specific diagnostic codes in the clinic before surgery, e.g., unspecified monoarthritis (ICD-9-CM: 716.6); other unspecified arthropathy (716.9); pain in the joint, pelvic region, and thigh; acute pain and chronic pain (338*); sprains and strains of the hip and thigh (ICD-9-CM: 843); and arthralgia of the hip (719.45), and these diagnoses were not captured for analysis. This may explain why the rates of hip- and spine-related diagnoses before surgery were not the same as the diagnosis rates at discharge.

## 5. Conclusions

The hip and spine can create similar patterns of pain and dysfunction. The purpose of this study was to assess whether orthopedic surgeons or neurosurgeons assessed and diagnosed hip-spine syndrome more frequently. We also hope to increase awareness of the coexistence of degenerative spine and hip diseases and the importance of preoperative assessments and radiographic examinations for both of these conditions. Using the Taiwan NHIRD, we demonstrated that orthopedic surgeons may obtain more hip imaging and diagnose more hip conditions before spinal surgery than neurosurgeons.

## Figures and Tables

**Table 1:** Demographics, length of stay, and Charlson Comorbidity Index (CCI) for each group.

Characteristics	Hip and Spine Surgery in the Same Admission (Both) (*n* = 102)		Hip Before Spine Surgery (HS) (*n* = 414)		Spine Before Hip Surgery (SH) (*n* = 1276)	
Age	65.9 (7.8)		66.8 (8.4)		66.9 (7.9)	
1st hospital stays	17.8 (10.3)		8.8 (4.6)		9.8 (5.7)	
1st + 2nd hospital stays	17.8 (10.3)		20.2 (11.9)		17.8 (7.3)	
Duration between admissions	0		189.6 (98.1)		162.6 (93.5)	
CCI at 1st admission	1.9 (2.7)		1.8 (2.4)		1.6 (2.3)	
CCI at 2nd admission			1.8 (2.3)		1.6 (2.4)	
	*n*	%	*n*	%	*n*	%
Female	65	63.7	262	63.3	810	63.5
Male	37	36.3	152	36.7	466	36.5

All number presented as mean (standard deviation), except sex.

**Table 2:** Comparison of the numbers of surgeries performed by orthopedic surgeons and neurosurgeons.

	Hip and Spine Surgery at the Same Admission (Both) (*n* = 102)		Hip Before Spine Surgery (HS) (*n* = 414)		Spine Before Hip Surgery (SH) (*n* = 1276)	
	*n*	%	*n*	%	*n*	%
First surgery by orthopedic surgeon	81	79.4	399	96.4	679	53.2
First surgery by neurosurgeon	3	2.9	0	0.00	522	40.9
Second surgery by orthopedic surgeon	-	-	264	63.8	1256	98.4
Second surgery by neurosurgeon	-	-	112	27.1	0	0.00
Orthopedic clinic visit before first surgery (<6 months)	85	83.3	354	85.5	863	67.6
Neurosurgery clinic visit before first surgery (<6 months)	14	13.7	42	10.1	460	36.1
Orthopedic and neurosurgery clinic visits before first surgery	8	7.8	37	8.9	229	18.0

**Table 3:** Comparison of preoperatively arranged imaging studies in each group.

Characteristics	Hip and Spine Surgery at the Same Admission (Both) (*n* = 102)		Hip Before Spine Surgery (HS) (*n* = 414)		Spine Before Hip Surgery (SH) (*n* = 1276)	
	*n*	%	*n*	%	*n*	%
Had spinal AP and spine lateral X-rays before first surgery	87	85.3	249	60.1	1048	82.1
Had KUB X-ray before first surgery	8	7.8	84	20.3	302	23.7
Had pelvic X-ray before first surgery	73	71.6	336	81.2	445	34.9

AP, anteroposterior; KUB, kidney/ureter/bladder.

**Table 4:** Comparison of the diagnoses of spine or hip disorders in each group.

Characteristics	Hip and Spine Surgery at the Same Admission (Both) (*n* = 102)		Hip Before Spine Surgery (HS) (*n* = 414)		Spine Before Hip Surgery (SH) (*n* = 1276)	
	*n*	%	*n*	%	*n*	%
Had spinal disease-related diagnosis before surgery	67	65.7	215	51.9	963	75.5
Had hip osteoarthritis diagnosis before surgery	54	52.9	264	63.8	480	37.6
Had hip osteonecrosis diagnosis before surgery	5	4.9	68	16.4	44	3.5
Had spine-related-diagnosis at first discharge	95	93.1	51	12.3	1260	98.8
Had hip-related diagnosis at first discharge (osteoarthritis)	91	89.2	376	90.8	145	11.4
Had hip-related diagnosis at first discharge (osteonecrosis)	25	24.5	144	34.8	52	4.1

**Table 5:** Comparison of preoperative imaging studies.

	Spine Surgery Was Performed by OS (*n* = 679)	Spine Surgery Was Performed by NS (*n* = 522)	*p*-Value
Preoperative spinal X-ray (32011C or 32012C) <6 months	566 (83.4)	420 (80.5)	*p* = 0.1941
Preoperative spine X-ray + KUB or pelvic X-ray {32022C or 32006C and (32011C or 32012C)} <6 months	357 (52.6)	199 (38.1)	*p* < 0.001

OS, orthopedic surgeon; NS, neurosurgeon; KUB, kidney/ureter/bladder. Data are the number (percentage).

**Table 6:** Comparison of diagnoses of spinal or hip disorders.

	Spine Surgery Was Performed by OS (*n* = 679)	Spine Surgery Was Performed by NS (*n* = 522)	*p*-Value
Spine-related diagnosis before surgery	491 (72.3)	415 (79.5)	*p* = 0.0041
Hip-related diagnosis before surgery	296 (43.6)	151 (28.9)	*p* < 0.001

OS, orthopedic surgeon; NS, neurosurgeon.

## Data Availability

The data presented in this study are available upon reasonable request from Dr. Wei-Cheng Lo (nicholaslo@tmu.edu.tw).

## Appendix A

**Table A1:** Summary of spine, hip arthroplasty, and radiology procedure codes used to select patients from the Taiwan NHIRD.

**Included procedural codes**	**Description**
64162B	Total hip arthroplasty
64170B	Partial hip arthroplasty
83002C	Laminectomy for decompression of ≤2 segments
83003C	Laminectomy for decompression of >2 segments
83024C	Discectomy—lumbar region
83043B	Spinal fusion—anterior spinal fusion without spinal instrumentation
83044B	Spinal fusion—anterior spinal fusion with spinal instrumentation
83045B	Spinal fusion—posterior spinal fusion without spinal instrumentation
83046B	Spinal fusion—posterior spinal fusion with spinal instrumentation ≤6 motion segments
**Excluded procedural codes**	**Description**
64029B	Open reduction for fracture of femoral neck or intertrochanteric fracture
83022C	Discectomy—cervical region
64236B	Open reduction of acetabulum fracture
64204B	Wide excision of malignant bone tumor
64206B	Excision of benign bone tumor
64042C	Closed reduction of fracture of spine of pelvis bone
64160B	Open reduction of fracture of spine
64164B	Open reduction of fracture of pelvis
33126B	Vertebroplasty or kyphoplasty
64005B	Sequestrectomy or saucerization and debridement for osteomyelitis
48004C–48006C	Debridement of the wound
**Radiographic procedural codes**	**Description**
32011C or 32012C	L-spine X-ray examination (AP and lateral views)
32022C	AP pelvic or AP and lateral hip X-ray
32006C	KUB X-ray

NHIRD, National Health Insurance Research Database; AP, anteroposterior; KUB, kidney/ureter/bladder.

**Table A2:** Summary of excluded ICD-9 diagnosis codes.

Code	Description
820 *	Hip fracture
805 *; 806 *	Fracture of vertebral column without/with mention of spinal cord injury
723 *	Other disorders of the cervical region
170 *	Malignant neoplasm of bone and articular cartilage
198 *	Secondary malignant neoplasm
015 *	Tuberculosis of bones and joints
730	Osteomyelitis periostitis and other infections involving bone
711 *	Arthroplasty-associated infections
324 *	Intracranial or intraspinal abscess
996–999	Procedure- and medical care-related complications

* Including all subgroups.

**Table A3:** Lumbar spine- and hip-related ICD-9 diagnosis codes.

**Spine-related codes**	**Description**
720 *	Ankylosing spondylitis and other inflammation spondylopathies
721.3	Lumbosacral spondylosis without myelopathy
721.42	Lumbar spondylosis with myelopathy
721.9	Spondylosis with unspecified site
722.10	Displacement of lumbar intervertebral disc without myelopathy
722.2	Displacement of intervertebral disc without myelopathy, site unspecified
722.32	Schmorl’s nodes, lumbar region
722.52	Degeneration of lumbar or lumbosacral intervertebral disc
722.6	Degeneration of intervertebral disc, site unspecified
722.73	Intervertebral disc disorder with myelopathy, lumbar region
722.93	Other unspecified disc disorder, lumbar region
724.02–724.03	Spinal stenosis, lumbar region
724.2	Lumbago
724.3	Sciatica
724.5–724.9	Backache
756.11	Spondylolysis, lumbosacral region
756.12	Spondylolisthesis
**Hip-related codes**	**Description**
71515	Osteoarthrosis, pelvic region and thigh, and primary
71525	Osteoarthrosis, pelvic region and thigh, and secondary
71535	Osteoarthrosis localized not specified whether primary or secondary involving pelvic region and thigh
71595	Osteoarthrosis, unspecified whether generalized or localized, and pelvic region and thigh
73342	Aseptic necrosis of head and neck of femur

* Including all subgroups.
